# Uterine Hemorrhage, with Expulsion of the Ovum, Successfully Treated

**Published:** 1842-03

**Authors:** J. H. Holmes

**Affiliations:** Spring Ridge, Mississippi


Art. II.—*Uterine Hemorrhage, with expulsion of the Ovum, successfully treated.* By Dr. J. H. Holmes, of Spring Ridge, Mississippi.

I was called on the 13th day of August 1839, in consultation, to see Mrs. H., aged 30, of sanguine temperament, and in the third month of pregnancy. I learned that she had received a fall from a horse in the early part of the day, one or two hours after which she complained of severe pain in the back and loins, occasionally extending to the uterus; these pains continued for some time, accompanied by a profuse discharge of blood from the vagina.

The midwife who had been called to see Mrs. H., becoming alarmed at her situation, had Dr. F. sent for, who, on his arrival, immediately bled the patient, gave her an anodyne, and ordered cold applications to the abdomen. These means for a time arrested the discharge; finally it returned with more violence than ever, and he ordered a pill of the acetate of lead and opium every hour. An examination was now made to learn the situation of the os tincae; this was found considerably relaxed and dilated.

At 3 o'clock, P. M., the flooding was violent, accompanied by trifling pain, frequent syncope, and a very small, quick pulse. At the suggestion of Dr. F., I was sent for from a distance of twelve miles. When I arrived, at 9 o'clock P. M., I could find no pulse at the wrist; the flooding continued slowly, with complete atony of the uterus; there was frequent syncope and vomiting, and occasionally delirium. I inquired of Dr. F. and the midwife, if the ovum had been expelled; they were confident that it had not come away. I was not disposed to make an examination myself in regard to the situation of the os tincae, from the fact that frequent touching is not only injurious by fatiguing the patient, but by removing the coagula that may be important in stopping the hemorrhage.

I advised the free administration of ergot in decoction. We had some prepared in a few minutes and given to her, but the stomach instantly rejected it. Alkalies and absorbents were then resorted to, and a sinapism was applied over the stomach with a view of quieting it. The ergot was again tried with a hope that it would be retained; but it seemed that we were only to meet with disappointment on all sides.

I suggested the propriety of making Mrs. H.'s situation known to her husband, and urging a speedy delivery; this was done by Dr. F., he being the family physician. The reply was, that he would rather see her die than that we should make an effort of that kind. The ergot, acetate of lead, opium, and cold applications having failed, we had but one alternative left, that of using the tampon, and leaving nature to effect a delivery. However, in the midst of this dilemma, we proposed to Mrs. H. to allow us to rupture the membranes and thus bring about a speedy delivery; to this she readily consented.

I was selected to make the examination, and see what could be done. Introducing the index finger of the right hand, I found the os externum very much relaxed, and the os tincae considerably dilated; upon extending the finger beyond the os tincae, I discovered that the placenta was loose and flabby, but still adherent to the fundus and right side of the uterus. I also convinced myself that the ovum had been discharged at some time during the early part of the day. I endeavored to get hold of the placenta between the finger and thumb, and extract it, but having failed in this we procured a small wire, bent at one end in the shape of a hook. The curved extremity of the wire was conducted along the finger of the right hand to the fundus of the uterus, and being drawn down gently, was hooked into the placenta, which it brought away. A tampon of old linen previously oiled was introduced with a view of arresting any further hemorrhage (for at this moment every ounce of blood was of immense value to the patient), until a contraction of the womb came on. Her complete exhaustion, cold extremities, and frequent syncope, induced us to apply a roller of flannel, dipped in warm turpentine, to her extremities. In two hours a partial reaction had ensued, and by morning we had the pleasure of seeing our patient so far improved as to consider her in a fair way to recover with care. The tampon was extracted next evening; her recovery was very slow and gradual, being confined to her room for four weeks.

I have been induced to report this case, not for any thing novel in its treatment or management, but for its rare occurrence, and the success that attended our imperfect wire crotchet.

September, 1841.

